# Laparoscopic versus open major liver resection for hepatocellular carcinoma: A case-matched analysis of short- and long-term outcomes

**DOI:** 10.1515/med-2021-0308

**Published:** 2021-06-30

**Authors:** Aoxiao He, Zhihao Huang, Jiakun Wang, Qian Feng, Rongguiyi Zhang, Hongcheng Lu, Long Peng, Linquan Wu

**Affiliations:** Department of General Surgery, The Second Affiliated Hospital of Nanchang University, Nanchang 330006, Jiangxi, China; Department of Emergency, The Second Affiliated Hospital of Nanchang University, Nanchang 330006, Jiangxi, China

**Keywords:** lapaproscopic, open, hepatocellular carcinoma, case-matched analysis, liver resection

## Abstract

**Background:**

The feasibility and safety of laparoscopic major hepatectomy (LMH) are still uncertain. The purpose of the present study is to compare the short- and long-term outcomes of LMH with those of open major hepatectomy (OMH) for hepatocellular carcinoma (HCC).

**Method:**

Between January 2012 and December 2018, a total of 26 patients received laparoscopic major hepatectomy in our center. To minimize any confounding factors, a 1:3 case-matched analysis was conducted based on the demographics and extent of liver resection. Data of demographics, perioperative outcomes, and long-term oncologic outcomes were reviewed.

**Results:**

Intraoperative blood loss (*P* = 0.007) was significantly lower in the LMH group. In addition, the LMH group exhibited a lower overall complication rate (*P* = 0.039) and shorter postoperative hospital stay (*P* = 0.024). However, no statistically significant difference was found between LMH and OMH regarding operation time (*P* = 0.215) and operative cost (*P* = 0.860). Two laparoscopic cases were converted to open liver resection. In regard to long-term outcomes, there was no significant difference between LMH and OMH regarding disease-free survival (DFS) (*P* = 0.079) and overall survival (OS) (*P* = 0.172).

**Conclusion:**

LMH can be an effective and safe alternative to OMH for selected patients with liver cancer in short- and long-term outcomes.

## 1 Introduction

Laparoscopic liver resection (LLR) has been increasingly utilized by surgeons since the first introduction of LLR in 1992 [1]. With the continuous development in laparoscopic devices and approaches, laparoscopic minor resections have even become standard surgical procedures for treating solitary lesions located in liver segments 2–6 [2,3,4]. However, laparoscopic major hepatectomy (LMH) has been relatively slow because LMH often correlated with a high risk of uncontrollable intraoperative bleeding, difficult procedures, and high rate of conversion. The second International Consensus Conference of Morioka recommended that LMH comprised innovative procedures in the exploration phase and could be performed only by those with experience of major open liver resections and advanced laparoscopic techniques [4]. With accumulating the development of new instruments, the introduction of novel techniques, the improvements in surgical skills, and experience of LLR, some recent studies reported that LMH and OMH had similar oncologic outcomes in patients with hepatocellular carcinoma (HCC) [5,6,7,8]. However, just a few studies described the long-term oncologic outcomes of LMH for HCC. Therefore, the present study aimed to compare the short- and long-term outcomes between LMH and OMH in patients with hepatic disease, especially those with HCC.

## 2 Materials and methods

### 2.1 Patients

Between January 2012 and December 2018, we retrospectively collected data of 26 patients who underwent LMH for HCC at The Second Affiliated Hospital of Nanchang University. In addition, a 1:3 case-matched analysis of patients (*n* = 78) who underwent open major hepatectomy (OMH) was also conducted based on the demographics and extent of liver resection. The study protocol was approved by the Institutional Review Board at the Second Affiliated Hospital of Nanchang University, and the informed consents were obtained from all patients. Data of the medical records including patient demographics, perioperative outcomes, and long-term oncologic follow-up were retrieved. The patients were divided into two groups according to the type of procedure: LMH group (*n* = 26) and OMH group (*n* = 78). The study was conducted in accordance with the Helsinki Declaration of 1964 and all subsequent amendments, and it was approved by the Ethics Committee of the Second Affiliated Hospital of Nanchang University in China, and all patients provided written informed consent.

### 2.2 Definitions

According to The Brisbane 2000 terminology, major hepatectomy (MH) was defined as resection of more than three liver segments [9,10]. Because the right posterior sectionectomy and the right anterior sectionectomy were difficult to perform by open laparoscopic hepatectomy, these procedures were also considered as MH [11]. The overall complication was defined as those that occurred within 30 days after hepatectomy. The Claviene–Dindo classification was used to grade the severity of complications [12]. Postoperative mortality was defined as death within 90 days after surgery.

### 2.3 Surgical procedures

The preferred type of liver resection was anatomical resection, if indicated. The selection for the type of liver resection was based on the remaining liver function, the proximity of lesions to major vascular structure, the number of lesions, and the depth of the lesion. If the hepatic reserve was expected to be enough for the deep-seated lesion, major liver resection was performed. The hepatic reserve was evaluated in terms of the computed tomographic volumetry and indocyanine green retention rate at 15 min (ICG-R15). The indication of LMH was similar to that of OMH, including the terms of the hepatic reserve, type of hepatectomy, and postoperative care [13,14]. In patients with central lesions in the suprahepatic junction adjacent to the major hepatic vein and tumors adjacent or invading to the main portal pedicle or inferior vena cava, however, laparoscopic hepatectomy was not usually considered.

**Figure 1:**
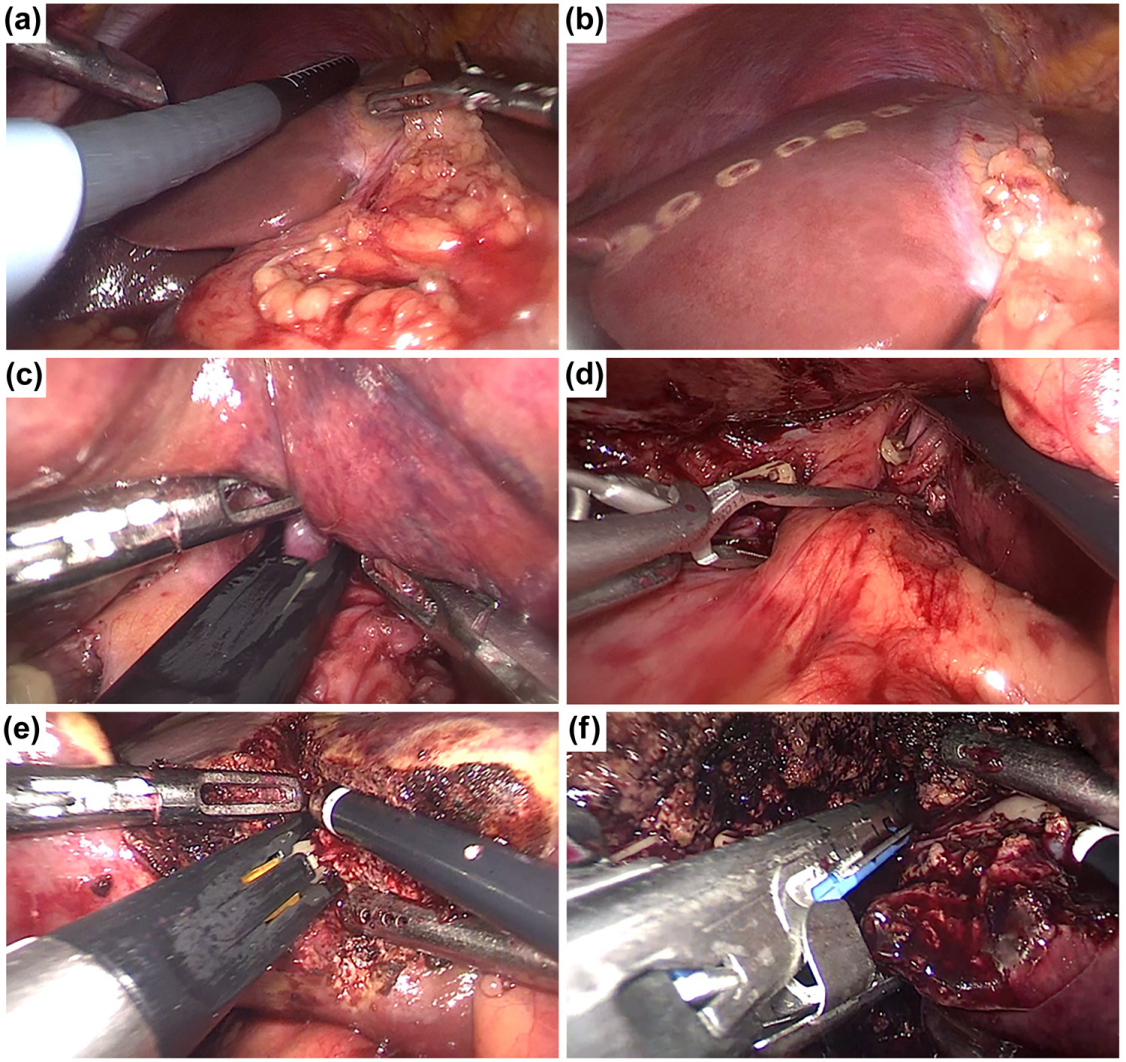
Surgical techniques for LMH. (a and b) intraoperative ultrasonography is used routinely and the hepatic transection line was marked. (c and d) The Glissonean approach is used to control the liver inflow. (e and f) The left portal vein and hepatic artery are isolated and divided by Hem-o-lock clips and/or Endo-GIA device.

It has been described in more detail elsewhere for the techniques of LMH and OMH performed at our institution [13,14]. For both anatomical right or left hepatectomy, intraoperative ultrasonography was used routinely to decide the type of hepatectomy and get the free resection margin (Figure 1a and b). The Glissonean approach was used to control the liver inflow before mobilization of the hepatic lobe (Figure 1c and d). For right posterior sectionectomy or hemihepatectomy, multiple small hepatic veins were divided, and the liver was fully mobilized from the inferior vena cava as much as possible. For left hemihepatectomy, the round ligament was first divided. Then, the left triangular ligaments and left falciform were dissected until the left hepatic vein was exposed. The left portal vein and hepatic artery were isolated and divided by Hem-o-lock clips and or Endo-GIA device, after fully mobilizing the left liver (Figure 1e and f).

### 2.4 Postoperative care and follow-up

The same postoperative monitoring and care were given to all patients, which included routine liver function tests and blood examinations. The abdominal drainage was removed in the absence of bile leakage or peritonitis. Assessment of serum AFP levels, ultrasonography, CT, and liver function tests was required bimonthly during the first postoperative year of follow-up. Then, the aforementioned tests should be required quarterly if no recurrence was detected. Recurrence was defined as HCC, characteristic findings on follow-up CT or MRI.

### 2.5 Statistical analysis

SPSS 17.0 software (IBM Inc., Chicago, IL, USA) was used to process all data. Categorical variables were compared using *χ* ^2^ test or Fisher’s exact test as appropriate, and continuous parameters using Student’s *t* test. Kaplan–Meier estimates for DFS and OS were compared between the LMH group and the OMH group using the log-rank test. *P* < 0.05 was regarded as statistically significant.

## 3 Results

During the study period, a total of 456 consecutive patients were treated by hepatectomy. Of these patients, 26 patients underwent LMH, and a 1:3 case-matched analysis of patients (*n* = 78) who underwent OMH was also conducted based on the demographics and extent of liver resection.

### 3.1 Patients’ characteristics

Patients’ characteristics of both groups are listed in Table 1. No significant differences were found between both groups in preoperative demographic characteristics, including gender, age, BMI, Child–Pugh classification, histologic cirrhosis, comorbidities, tumor size, and the number of tumors.

**Table 1:** Patients’ characteristics and perioperative outcomes

	LMH (*n* = 26)	OMH (*n* = 78)	*p* value
Gender (M:F)	11:15	33:45	1.000
Age (years)	56.1 ± 10.6	52.0 ± 12.2	0.698
BMI (kg/m^2^)	23.8 ± 3.0	22.8 ± 2.7	0.110
**Child–Pugh class, *n* (%)**			
A	23 (88.5)	70 (89.7)	1.000
B	3 (11.5)	8 (10.3)	
Histologic cirrhosis	16 (61.5)	45 (57.7)	0.730
**Comorbidities, *n* (%)**			
Diabetes	2 (7.6)	7 (9.0)	1.000
Hypertension	4 (15.4)	8 (10.26)	0.489
Underlying hepatic disease	11 (42.3)	29 (37.2)	0.642
Tumor size (mm)	75.0 ± 35.1	75.5 ± 38.8	0.378
Number of tumors	1.3 ± 0.6	1.4 ± 0.7	0.381

Continuous variables in the table are expressed as mean ± SD.

LMH, laparoscopic major hepatectomy; OMH, open major hepatectomy; M, male; F, female.

### 3.2 Surgical results

The surgical results of both groups are listed in Table 2, and no mortality during surgery was observed. Two patients (7.7%) in the laparoscopic group were converted to open procedure because of uncontrollable bleeding during parenchymal transection. A total of 55 patients (70.5%) in the OMH group and 15 patients (57.7%) in the LMH group (*P* = 0.227) were treated by portal triad clamping during hepatectomy. Significantly less intraoperative blood loss was found in the LMH group than in the OMH group (340.8 ± 225.2 mL vs 601.4 ± 509.4 mL, *P* = 0.007); however, no significant difference between the LMH group and the OMH group was found in intraoperative transfusion (26.9 vs 29.5%, *P* = 0.803). In addition, the operation time did not differ significantly between both groups (264.2 ± 14.1 min vs 255.4 ± 36.3 min, *P* = 0.215).

**Table 2:** Operative outcomes

	LMH (*n* = 26)	OMH (*n* = 78)	*P* value
Operation time (min)	264.2 ± 14.1	255.4 ± 36.3	0.215
Intraoperative blood loss (mL)	340.8 ± 225.2	601.4 ± 509.4	**0.007**
Intraoperative transfusion, *n* (%)	7 (26.9)	23 (29.5)	0.803
Total complication, *n* (%)	4 (15.4)	29 (37.2)	**0.039**
Wound infection	2 (7.7)	6 (7.7)	1.000
Bile leakage	1 (3.8)	5 (6.4)	1.000
Intraabdominal fluid collection	1 (3.8)	10 (12.8)	0.357
Bleeding, *n* (%)	0	1 (1.3)	1.000
Pulmonary infection, *n* (%)	0	5 (6.4)	0.427
Abdominal incisional hernia	0	2 (2.6)	1.000
Recovery of bowel movement, days	1.5 ± 0.5	3.1 ± 0.6	0.083
Time of off-bed activities, days	2.8 ± 0.6	4.9 ± 1.1	**0.003**
Postoperative hospital stay, days	11.0 ± 2.9	15.5 ± 5.2	**0.024**
pR1, *n* (%)	1 (3.8)	7 (9.0)	0.671
pRM (mm)	7.5 ± 35.1	7.1 ± 36.4	0.895
Operative cost (RMB)	4850.0 ± 1041.8	4790.3 ± 904.3	0.860
Overall cost (RMB)	56306.4 ± 9477.5	59251.9 ± 16075.6	**0.024**

Continuous variables in the table are expressed as mean ± SD.

LMH laparoscopic major hepatectomy, OMH open major hepatectomy, pR1 positive surgical resection margin, pRM pathological resection margin.

*P* < 0.05 was considered statistically significant and was shown in bold.

A total 18 patients (69.2%) in the LMH group and 54 patients (69.2%) in the OMH underwent right hepatectomy. Pathologic examination of free resection margin was similar between both groups (96.2 vs 91.0%, *P* = 0.671).

### 3.3 Postoperative outcomes and cost

Postoperative results of both groups are listed in Table 2. There were one laparoscopy patient (3.8%) and five (6.4%) patients undergoing open surgery with hepatectomy-related complications after surgery (*P* = 1.000). Overall complications were significantly lower in the LMH group compared to the OMH group (15.4 vs 37.2%, *P* = 0.039). There was no perioperative mortality between both groups. Although no significant difference was found in the recovery of the bowel movement (1.5 ± 0.5 days vs 3.1 ± 0.6 days, *P* = 0.083) between both groups, duration of off-bed activities (2.8 ± 0.6 days vs 4.9 ± 1.1 days, *P* = 0.003) and postoperative hospital stay (11.0 ± 2.9 days vs 15.5 ± 5.2 days, *P* = 0.024) was significantly shorter in the LMH group compared to the OMH group. Both the surgical and overall costs were collected. Interesting, we found that although no significant difference was found in surgical cost between both groups (4850.0 ± 1041.8 RMB vs 4790.3 ± 904.3 RMB, *P* = 0.860), the overall cost of the LMH group was significantly lower than the OMH group (56306.4 ± 9477.5 RMB vs 59251.9 ± 16075.6 RMB, *P* = 0.024).

### 3.4 Long-term survival outcomes

**Figure 2:**
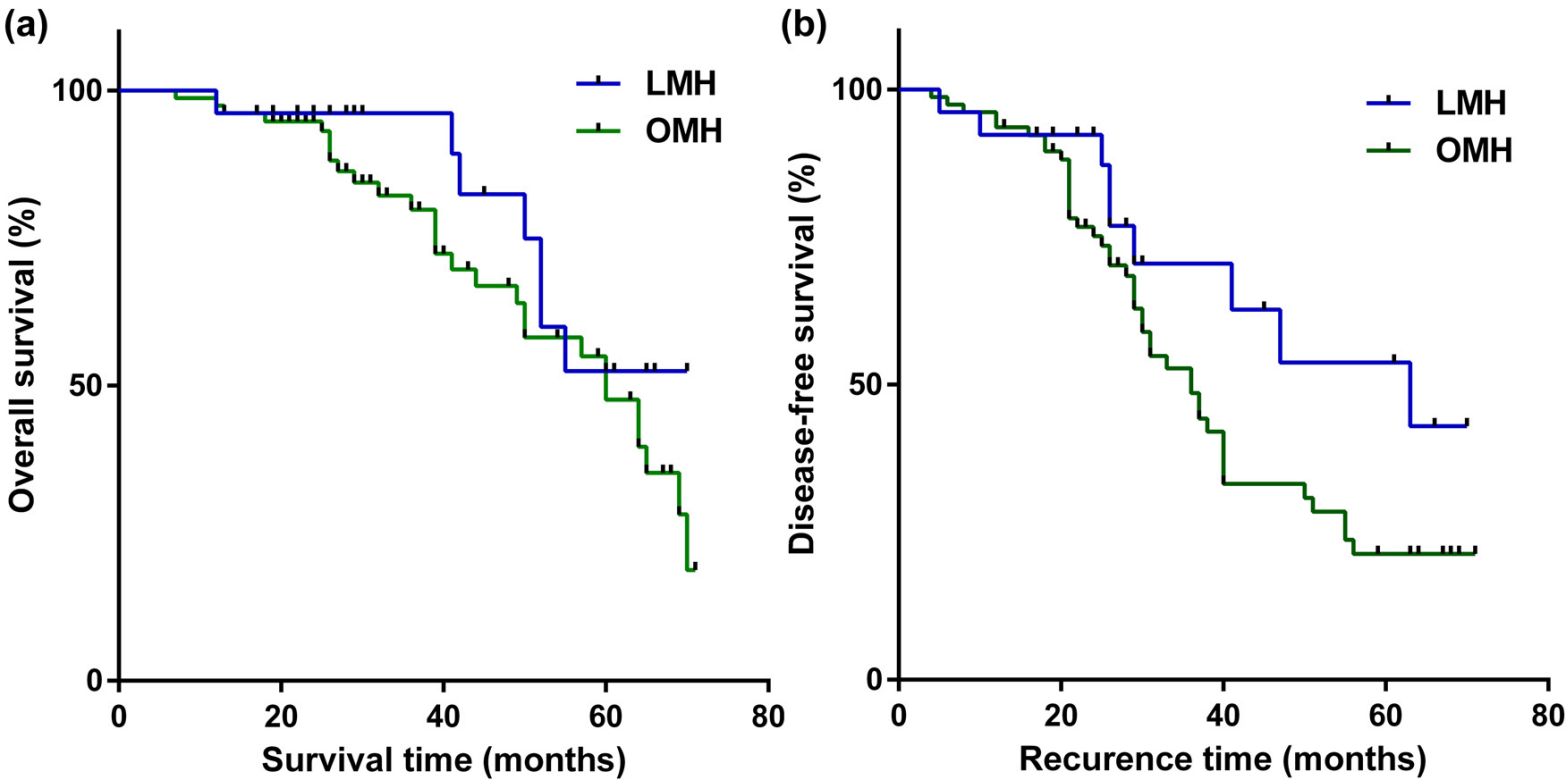
Weighted Kaplan–Meier plot for DFS and OS for LMH versus OMH. (a) Median DFS of LMH and OMH groups was 63.0 and 36.0 months (*P* = 0.079), respectively. (b) Median OS of LMH and OMH groups was 60.0 and 60.0 months (*P* = 0.172), respectively.

The follow-up was 33.3 ± 15.6 months in the LMH group and 31.4 ± 15.7 months in the OLH group, and no significant difference was found between both groups (*P* = 0.752). The median OS of LMH and OMH groups was 60.0 months (95% CI, 50.3–69.7 months) and 60.0 months, respectively (95% CI, 47.6–72.4 months; Figure 2a). The median DFS of LMH and OMH groups was 63.0 months (95% confidence interval [CI] 31.8–94.1 months) and 36.0 months (95% CI, 29.7–42.3 months), respectively (Figure 2b). No significant difference in OS (*P* = 0.172) and DFS (*P* = 0.079) was found between both groups.

## 4 Discussion

With the continuous development in laparoscopic devices and approaches, laparoscopic minor liver resections have even become standard surgical procedures for treating solitary lesions located in liver segments 2–6 [2,3,4]. Due to the long learning curve for LLR, it is necessary to consider the expertise of the surgeon for safe laparoscopic minor resection [15,16]. Recently, in some highly specialized centers, LMH can be performed as effectively and safely as OMH [3]. LMH even was not inferior to OMH in terms of resection margin, postoperative complications, operative mortality, and long-term outcomes stated by the Second International Consensus Conference held in Morioka; in addition, LLR was superior in terms of shorter hospital stay [4].

As presented in Table 3, we have summarized all comparative studies of major LLR vs major OLR [4,5,6,7,8,17,18,19,20,21,22,23]. The negative margins and oncologic integrity of the procedure should be obtained, when major LLR is performed for cancer. No difference in the resection margin was found in the comparative studies of major LLR vs major OLR, although the tumor size of major LLR was large than that of major OLR in the studies by Guro et al. [8], Goumard et al. [17], Komatsu et al. [18], and Tarantino et al. [20]. In the present case-matched study, the negative margin of major LLR was similar to major OLR. In addition, the R0 resection rate of the LMH group was 96.2%. Recently, some meta-analyses of retrospective studies also observed that no significant difference was found between major LLR and major OLR in the resection margin for HCC patients [24,25]. To better learn major LLR for HCC patients, long-term survival rate should also be obtained. As shown in Table 3, data of 5-year over survival (OS) and disease-free survival (DFS) were obtained from three studies including the data of our study. Although the laparoscopic group has a longer OS compared to open group, no significant difference was found between both groups with regard to OS and DFS. In addition, Wang et al. [26] conducted a meta-analysis, which compared short- and long-term outcomes of major LLR with those of major OLR. The results of this meta-analysis showed that major LLR had advantages in intraoperative blood loss, postoperative hospital stay, and postoperative morbidity. Therefore, we can conclude that major LLR may be as oncological safety as major OLR. Because the aforementioned data come from observational clinical studies, however, additional randomized controlled trials are required to provide convincing evidence in the future.

**Table 3:** Main characteristics of the comparative studies of major LLR vs major OLR

Authors	Type of surgery	No. of patients	Tumor size (mm)	Number of tumors	Resection margins (mm)	Operation time (min)	Intraoperative blood loss (mL)	No. transfusions	Total complications	Postoperative hospital stay (days)	Hospital death	OS	DFS
Chen et al. [5]	LMH	126	64 (14–130)	112/14	NA	**240 (75–590)**	**200 (20–2500)**	**6 (4.8%)**	28 (22.2%)	**6 (3–21)**	0 (0%)	NA	NA
	OMH	133	67 (16–240)	110/23	NA	**230 (100–495)**	**400 (50–2,000)**	**22 (16.5%)**	36 (27.1%)	**8 (4–46)**	1 (0.9%)	NA	NA
Cho et al. [6]	LRPS	24	37 ± 18	NA	3.0 ± 5.8	**567.4 ± 212.4**	NA	NA	2 (8.3%)	10.6 ± 4.8	NA	79.1% (5 years)	42.2% (5 years)
	ORPS	19	48 ± 25	NA	7.0 ± 5.0	**316.1 ± 63.0**	NA	NA	4 (21.1%)	11.1 ± 3.2	NA	77.7%	51.5%
Cho et al. [7]	LCH	20	26 (6–140)	NA	7 (0.1–40)	**388 (246–661)**	350 (100–1,300)	2 (10%)	6 (30%)	8 (5–24)	6 (30%)	NA	NA
	OCH	20	27 (10–82)	NA	6.5 (0.1–23)	**268 (98–412)**	400 (50–3,300)	1 (5%)	4 (20%)	10 (5–24)	4 (20%)	NA	NA
Goumard et al. [8]	LRH	16	**39 (2–85)**	1.5 (1–3)	13.5 (0–50)	**360 (240–450)**	150 (100–700)	1 (6.3%)	4 (25%)	**7 (5–11)**	0 (0%)	67.1 % (3-year)	NA
	ORH	16	**62 (0–250)**	1.5 (1–3)	6.5 (0–60)	**300 (240–390)**	100 (100–800)	0 (0%)	8 (50%)	**12 (7–25)**	1 (6.3%)	NA	NA
Guro et al. [17]	LMH	67	**41 ± 24**	NA	2 (2.4%)	**416.6 ± 166.9**	1543.3 ± 2641.8	29 (34.9%)	**17 (20.5%)**	**11.3 ± 8.3**	1 (1.5%)	77.3% (5 years)	50.8% (5 years)
	OMH	110	**63 ± 38**	NA	6 (5.4%)	**332.5 ± 105.4**	1248.1 ± 1402.8	45 (40.5%)	**43 (38.7%)**	**18 ± 21.4**	3 (2.7%)	60.2%	40.1%
Komatsu et al. [18]	LMH	38	**47.5 (23–180)**	19/19	6 (15.8%)	**365 (180–600)**	100 (20–900)	2 (5.2%)	**12 (31.6%)**	7.5 (3–51)	NA	73.4 % (3 years)	50.3 % (3 years)
	OMH	38	**85.0 (20–180)**	16/22	6 (15.8%)	**300 (210–425)**	80 (20–800)	1 (2.6%)	**23 (60.5%)**	10.0 (5–53)	NA	69.2%	29.7%
Rhu et al. [19]	LRPS	53	31 ± 18	47/6	13 ± 10	**381 ± 149**	NA	7 (13.2)	5 (9.4%)	8.9 ± 3.6	0	NA	NA
	ORPS	97	31 ± 17	89/8	12 ± 9	**220 ± 91**	NA	2 (2.1)	8 (8.3%)	10.2 ± 3.6	1 (1%)	NA	NA
Tarantino et al. [20]	LRPS	13	**27 ± 9**	1.0 ± 0.2	10 ± 3	234 ± 57	125 ± 80	NA	**2 (15.3%)**	**5.7 ± 3**	NA	NA	NA
	ORPS	51	**37 ± 23**	1.0 ± 0.2	10 ± 2	216 ± 73	208 ± 263	NA	**27 (52.9%)**	**10.7 ± 5**	NA	NA	NA
Yoon et al. [21]	LRH	33	33.1 **±** 16.5	1.1 **±** 0.2	26 **±** 21	**297 ± 113**	125.5 **±** 229	0 (0)	1 (3.03%)	**9.97 ± 3.02**	NA	85.1% (2 years)	100% (2 years)
	ORH	33	29.6 **±** 15	1.1 **±** 0.2	20 **±** 15	**176 ± 60**	132 **±** 178	0 (0)	7 (21.21%)	**13.94 ± 3.37**	NA	83.9%	88.8%
Zhang et al. [22]	LRH	35	67 ± 42	NA	35/0	**309 ± 108**	**293 ± 82.5**	NA	**0**	**9 ± 2**	NA	NA	NA
	ORH	42	59 ± 30	NA	42/0	**223 ± 110**	**433 ± 105.5**	NA	**21 (50%)**	**15 ± 3**	NA	NA	NA
Zhang et al. [23]	LLH	20	67 ± 42	NA	20/0	143 **±** 35.6	**180 ± 20.5**	NA	**0**	**7 ± 1**	NA	NA	NA
	OLH	25	59 ± 30	NA	25/0	137 **±** 29.8	**350 ± 45.3**	NA	**10 (40%)**	**12 ± 2**	NA	NA	NA
Our results	LMH	26	75 ± 35	1.3 ± 0.6	7.5 ± 35.1	264.2 ± 14.1	**340.8 ± 225.2**	7 (26.9%)	**4 (15.4%)**	**11.0 ± 2.9**	0 (0%)	53.7% (5 years)	52.4% (5 years)
	OMH	78	76 ± 39	1.4 ± 0.7	7.1 ± 36.4	255.4 ± 36.3	**601.4 ± 509.4**	23 (29.5%)	**29 (37.2%)**	**15.5 ± 5.2**	0 (0%)	47.6%	21.4%

Continuous variables in the table are expressed as mean ± SD or median(range) unless otherwise specified.

LCH, laparoscopic central hepatectomy; LRPS, laparoscopic right posterior sectionectomy; LRH, laparoscopic right hepatectomy; LLH, laparoscopic left hemihepatectomy.

Statistically significant results are shown in bold.

*P* < 0.05 was considered statistically significant and was shown in bold.

With regard to the data on perioperative outcomes, major LLR was associated with favorable intraoperative blood loss, total postoperative complications, and postoperative hospital stay in the summarized comparative studies. However, the operation time of major LLR was significantly longer than major OLR in most of the retrospective studies [4,5,6,7,8,17,18,19,21,22]. Recently, the Japanese National Clinical Database showed that major LLR was associated with less blood loss, a lower complication rate, and shorter hospital stay compared with major OLR [27]. Regarding short-term outcomes in the present study, the average operation time of major LLR group was longer than OLR group. However, major LLR group has a significantly lower intraoperative blood loss and postoperative complication rate and shorter postoperative hospital stay. This indicates that although major LLR is technically more difficult than OLR, major LLR is similar to major OLR in short-term outcomes. Furthermore, owing to its minimal invasiveness, major LLR facilitates earlier patient recovery. Interestingly, our results showed that although no significant difference was found in surgical cost between both groups, the overall cost of the LMH group was significantly lower than the OMH group, which might be related to fast recovery.

To the best of our knowledge, the present report was the first study that summarized the long-term survival rate of major LLR in patients with HCC. However, there were many limitations in this study. First, this was a retrospective study, which may introduce bias. Second, although there was no difference in the resection margin between the two groups, we preferred major OLR in patients with HCC close to the major Glisson pedicle or the inferior vena cava.

## 5 Conclusion

In conclusion, major LLR of HCC is feasible and safe with favorable short- and long-term outcomes, when performed in experienced centers.
